# Chronic Restraint Stress Inhibits the Response to a Second Hit in Adult Male Rats: A Role for BDNF Signaling

**DOI:** 10.3390/ijms21176261

**Published:** 2020-08-29

**Authors:** Paola Brivio, Giulia Sbrini, Giulia Corsini, Maria Serena Paladini, Giorgio Racagni, Raffaella Molteni, Francesca Calabrese

**Affiliations:** 1 Department of Pharmacological and Biomolecular Sciences, Università deglI Studi di Milano, 20133 Milan, Italy; paola.brivio@unimi.it (P.B.); giulia.sbrini@unimi.it (G.S.); giuliacorsini892@gmail.com (G.C.); giorgio.racagni@unimi.it (G.R.); 2 Department of Medical Biotechnology and Translational Medicine, Università degli Studi di Milano, 20133 Milan, Italy; maria.paladini@unimi.it (M.S.P.); raffaella.molteni@unimi.it (R.M.)

**Keywords:** chronic stress, restraint stress, BDNF, BDNF signaling

## Abstract

Depression is a recurrent disorder, with about 50% of patients experiencing relapse. Exposure to stressful events may have an adverse impact on the long-term course of the disorder and may alter the response to a subsequent stressor. Indeed, not all the systems impaired by stress may normalize during symptoms remission, facilitating the relapse to the pathology. Hence, we investigated the long-lasting effects of chronic restraint stress (CRS) and its influence on the modifications induced by the exposure to a second hit on brain-derived neurotrophic factor (BDNF) signaling in the prefrontal cortex (PFC). We exposed adult male Sprague Dawley rats to 4 weeks of CRS, we left them undisturbed for the subsequent 3 weeks, and then we exposed animals to one hour of acute restraint stress (ARS). We found that CRS influenced the release of corticosterone induced by ARS and inhibited the ability of ARS to activate mature BDNF, its receptor Tropomyosin receptor kinase B (TRKB), and their associated intracellular cascades: the TRKB-PI3K-AKT), the MEK-MAPK/ERK, and the Phospholipase C γ (PLCγ) pathways, positively modulated by ARS in non-stressed animals. These results suggest that CRS induces protracted and detrimental consequences that interfere with the ability of PFC to cope with a challenging situation.

## 1. Introduction

Depression is a severe and highly diffuse disease affecting more than 10% of the general population, and by 2030, it is estimated to become the second leading cause of disability and the largest contributor to disease burden [1]. 

It has been largely characterized as a recurrent disorder, with approximately 50% of patients experiencing relapse [2]. Indeed, most of the remitted patients undergo recurrent episodes of depression in the 15 years after the first event of the pathology, with a persistence of residual symptoms [3,4].

Stress has been widely described as an environmental factor able to induce psychopathologies and, in particular, the depressive phenotype [5], and much work in animal modeling has relied on the observation that stress is a potent risk factor for the development of several psychiatric disorders. The effects of stress may depend on the intensity and duration of the adverse experience [6], as well as on the period of life when it begins. Indeed, negative life events occurring early in life may impact brain functioning and are critical for later psychopathologies [7], whereas the effect of stress in adulthood may have persistent consequences but may also recover physiologically through the activation of dynamic processes that help the brain to achieve successful adaptation [8]. However, limited information is available on the long-lasting impacts of stress exposure during adult life, as well as on the mechanisms that may promote or prevent relapse.

Moreover, it has been proved that exposure to stressful events may alter the response to subsequent stressors [9] possibly because not all the systems impaired by stress are restored during the remission, thus leaving ‘scars’ of vulnerability that may facilitate the relapse to the pathology.

Brain-derived neurotrophic factor (BDNF) is the most highly diffuse neurotrophin in the brain where it is involved in synaptic plasticity, as well as in neuronal function and survival during adulthood [10]. One of the hypotheses of depression proposes a role for BDNF in the etiology of the pathology [11,12], and it has been demonstrated that depressed patients have lower BDNF levels both in serum [13] and in the prefrontal cortex (PFC) and hippocampus (Hip) [14,15,16] with respect to healthy subjects. Accordingly, animal models useful for the study of stress-related disorders show a remarkable reduction in the neurotrophin mainly in the PFC and Hip [17,18,19].

The main purpose of this experiment was to investigate if and how stress-induced changes may persist beyond chronic stress exposure. To address this objective, we exposed adult male rats to four weeks of chronic restraint stress (CRS) and we left them undisturbed for the subsequent three weeks. After the period of washout, the animals were exposed to an acute restraint stress (ARS) in order to establish differences in responsiveness to the stressful event and to identify the long-lasting functional abnormalities that may render them more vulnerable to a challenging precipitating condition in the rest period.

At the molecular level, we aimed to clarify whether CRS may induce long-lasting changes in BDNF signaling and if the negative effect of stress may interfere with the ability to react with the subsequent ARS in terms of neuroplastic mechanisms. The analyses are focused on the PFC, the brain region involved in the stress response [20], characterized by low levels of BDNF in depressed patients [21] and known to be properly activated in terms of the markers of neuroplasticity in the timing of one hour after an ARS [22]

## 2. Results

### 2.1. Chronic Restraint Stress Induced Cognitive Impairment in Adult Male Rats

Exposure to 4 weeks of CRS induced a cognitive impairment as assessed by the novel object recognition (NOR) test. Indeed, as previously observed [23,24], we found a significant decrease in the NOR discrimination index in all the rats exposed to chronic stress (−32%, *p* < 0.05 vs. No stress, T value = 2.446) in comparison to non-stressed animals (Figure S1).

### 2.2. Chronic Restraint Stress Inhibited the Release of Corticosterone Levels Following the Acute Restraint Stress

**Figure 1:**
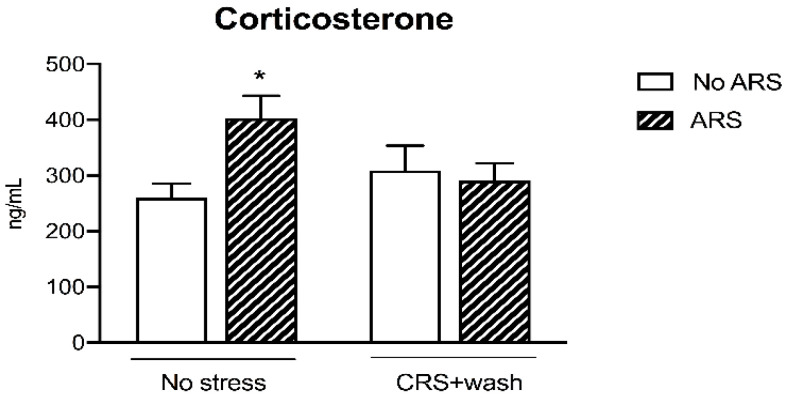
Analysis of corticosterone plasma levels in chronic restraint stress (CRS) rats exposed to an acute challenge (acute restraint stress (ARS)) after 3 weeks of washout (wash). The data are the mean ± SEM. * *p* < 0.05 vs. No stress/No ARS (two-way ANOVA with PLSD).

We then assessed the hypothalamic pituitary adrenal (HPA) axis responsivity to an acute challenge by measuring the corticosterone (CORT) plasma levels. As expected, the ARS induced an increase in CORT levels in non-stressed animals (+55%, *p* < 0.05 vs. No stress/No ARS, Fisher’s protected least significance difference (PLSD)), an effect that was completely blunted in animals previously exposed to CRS, as revealed by the significant CRSxARS interaction (F_1-38_ = 4.836, *p* < 0.05, two-way ANOVA) (Figure 1).

### 2.3. Chronic Stress Pre-Exposure Did Not Interfere with the Immediate-Early Genes Expression Following an Acute Restraint Stress

To evaluate if stress pre-exposure may alter the ability to respond to a challenging environmental stimulus, also after 3 weeks of rest from CRS, we investigated the expression of the immediate early genes (IEGs) *Arc*, *cFos*, and *Gadd45β*.

**Figure 2:**
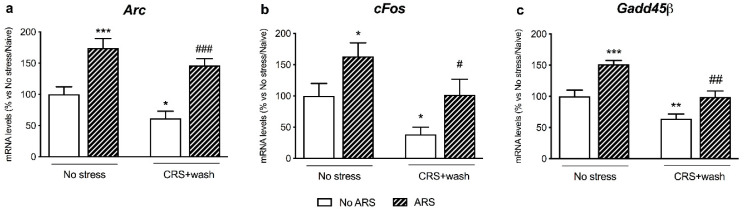
Analysis of *Arc* (panel **a**), *cFos* (panel **b**), and *Gadd45β* (panel **c**): mRNA levels in the prefrontal cortex of chronically stressed rats (CRS) exposed to an acute challenge (ARS) after 3 weeks of washout (wash). The data, expressed as a percent change of No stress/No ARS animals, are the mean ± SEM. * *p* < 0.05, ** *p* < 0.01, *** *p* < 0.001 vs. No stress/No ARS ; # *p* < 0.05, ## *p* < 0.01, ### *p* < 0.001 vs. CRS + washout/No ARS (two-way ANOVA with PLSD).

As shown in Figure 2, we found a significant effect of CRS (*Arc*: F_1-38_ = 6.872, *p* < 0.05; cFos: F_1–39_ = 9.536, *p* < 0.01; *Gadd45β*: F_1-40_ = 27.992, *p* < 0.001, two-way ANOVA) and of the ARS (Arc: F_1-38_ = 39.340, *p* < 0.001; *cFos*: F_1-39_ = 10.050, *p* < 0.01; *Gadd45β*: F_1-40_ = 26.904, *p* < 0.001, two-way ANOVA) on the IEGs expression. Indeed, we found a significant decrease in their mRNA levels in stressed animals after three weeks of washout (*Arc*: −39%, *p* < 0.05; cFos: −62%, *p* < 0.05; *Gadd45β*: −36%, *p* < 0.01 vs. No stress/No ARS, Fisher’s PLSD) in comparison to control animals. Moreover, independently from stress pre-exposure, the acute challenge induced a significant up-regulation in their gene expression in both non-stressed (Arc: +74%, *p* < 0.001; cFos: +63%, *p* < 0.01; *Gadd45β*: +51%, *p* < 0.001 vs. No stress/No ARS, Fisher’s PLSD) and stressed animals (*Arc*: +138%, *p* < 0.001; *cFos*: +167%, *p* < 0.05; *Gadd45β*: +55%, *p* < 0.01 vs. CRS + washout/No ARS, Fisher’s PLSD).

### 2.4. Chronic Stress Interfered with BDNF Signaling Following a Second Hit

In order to investigate whether stress-pre exposure may influence the activation of mature BDNF (mBDNF) signaling following a second hit, we measured the protein expression of the neurotrophin BDNF, of its high-affinity receptor TRKB, and their associated intracellular signaling cascades: The TRKB-PI3K-AKT, the MEK-MAPK/ERK, and the PLCγ pathways [25].

**Figure 3:**
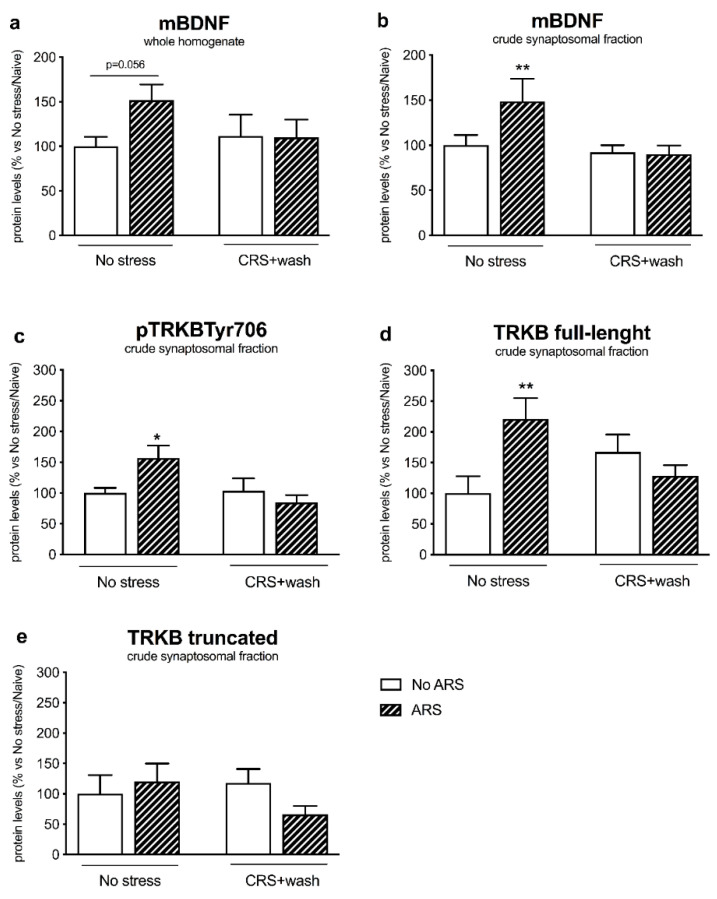
Analysis of mature brain-derived neurotrophic factor (mBDNF) (panels **a**,**b**) and TRKB receptor (phosphorylated, full-length and truncated form) (panels **c**–**e**) protein levels in the prefrontal cortex of chronically stressed rats (CRS) exposed to an acute challenge (ARS) after 3 weeks of washout (wash). The data, expressed as a percent change of No stress/No ARS animals, are the mean ± SEM. * *p* < 0.05, ** *p* < 0.01 vs. No stress/No ARS (two-way ANOVA with PLSD).

In the crude synaptosomal fraction, we observed a significant CRSxARS interaction (F_1–19_ = 7.535, *p* < 0.05, two-way ANOVA), with mBDNF protein levels being upregulated by the acute stress specifically in non-stressed animals (+49%, *p* < 0.01 vs. No stress/No ARS, Fisher’s PLSD) (Figure 3b), an effect that was paralleled by a trend of increase in the neurotrophin in the whole homogenate (+52%, *p* = 0.056 vs. No stress/No ARS, Fisher’s PLSD) (Figure 3a). Accordingly, as shown in Figure 3c, the restraint stress induced an increase in the phosphorylated form of the TRKB receptor in the Tyr706 (+57%, *p* < 0.05 vs. No stress/No ARS, Fisher’s PLSD) (CRSxARS interaction F_1-20_ = 4.687, *p* < 0.05, two-way ANOVA), an effect that was completely paralleled by the upregulation of the TRKB full-length form in control animals (+121%, *p* < 0.01 vs. No stress/No ARS, Fisher’s PLSD) but not in animals previously exposed to CRS (Figure 3d), whereas we did not observe any modulation for the truncated form of the receptor (Figure 3e). By contrast, in the whole homogenate (Table 1), we did not find a statistically significant effect for the CRS nor for the ARS on TRKB protein levels.

**Figure 4:**
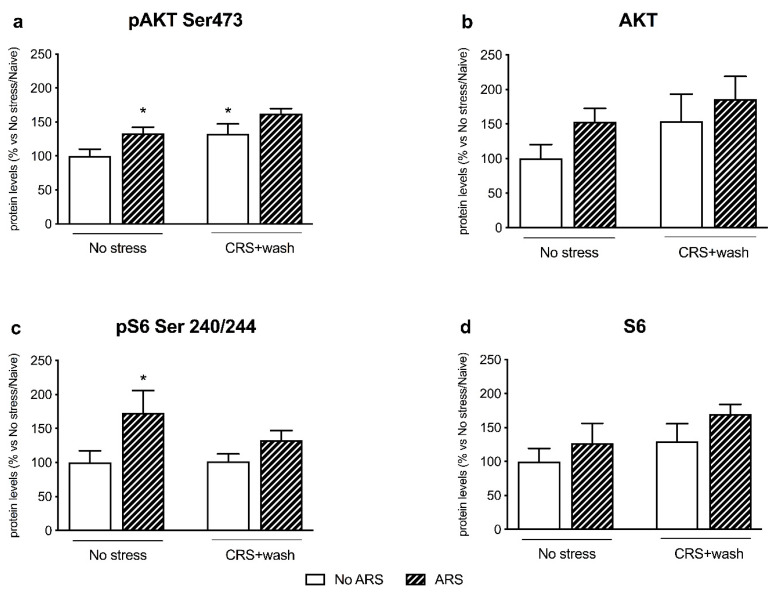
Analysis of AKT (panels **a**,**b**) and S6 (panels **c**,**d**) (phosphorylated and total) protein levels in the crude synaptosomal fraction of the prefrontal cortex of chronically stressed rats (CRS) exposed to an acute challenge (ARS) after 3 weeks of washout (wash). The data, expressed as a percent change of No stress/Naïve animals, are the mean ± SEM. * *p* < 0.05 vs. No stress/No ARS (two-way ANOVA with PLSD).

Regarding the TRKB-PI3K-AKT pathway, we observed, in the crude synaptosomal fraction, a significant effect of CRS (F_1–17_ = 7.836, *p* < 0.05, two-way ANOVA) and ARS (F_1–17_ = 8.168, *p* < 0.05, two-way ANOVA) on pAKT Ser473. Indeed, the pre-exposure to CRS induced an increase in its protein levels in naïve animals (+33%, *p* < 0.05 vs. No stress/No ARS, Fisher’s PLSD) (Figure 4a), whereas the acute challenge upregulated pAKT Ser473 expression only in non-stressed animals (+34%, *p* < 0.05 vs. No stress/No ARS, Fisher’s PLSD).

Accordingly, the phosphorylated form of one of the downstream effectors of AKT, pS6 Ser240/244, was significantly increased by the restraint stress in non-stressed animals (+73%, *p* < 0.05 vs. No stress/No ARS, Fisher’s PLSD), with an ARS effect (F_1–24_ = 6.329, *p* < 0.05, two-way ANOVA) (Figure 4c). However, the total forms of both AKT and S6 (Figure 4b–d respectively) were neither significantly modulated by CRS nor by ARS.

**Figure 5:**
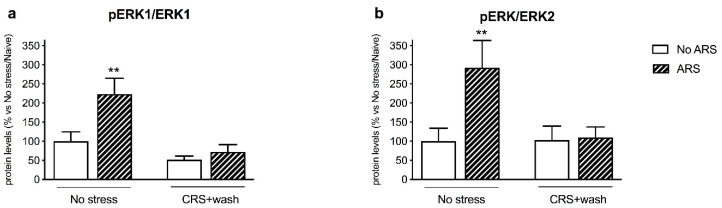
Analysis of ERK1/2 (panels **a**,**b**) (phosphorylated and total) protein levels in the crude synaptosomal fraction of the prefrontal cortex of chronically stressed rats (CRS) exposed to an acute challenge (ARS) after 3 weeks of washout (wash). The data, expressed as a percent change of No stress/Naïve animals, are the mean ± SEM. ** *p* < 0.01 vs. No stress/No ARS (two-way ANOVA with PLSD).

Moreover, the increased mBDNF levels coupled with the activation of TRKB induced a significant enhancement in pERK1/ERK1 (+123%, *p* < 0.01 vs. No stress/No ARS, Fisher’s PLSD) (CRS: F_1–16_ = 11.902, *p* < 0.01; ARS: F_1–16_ = 6.204, *p* < 0.05, two-way ANOVA) and pERK2/ERK2 (+192%, *p* < 0.05 vs. No stress/No ARS, Fisher’s PLSD) (ARS: F_1–16_ = 4.965, *p* < 0.05, two-way ANOVA) in the crude synaptosomal fraction, of the non-stressed group exposed to restraint stress, a modulation that was completely inhibited by the previous exposure to CRS (Figure 5a,b).

**Figure 6:**
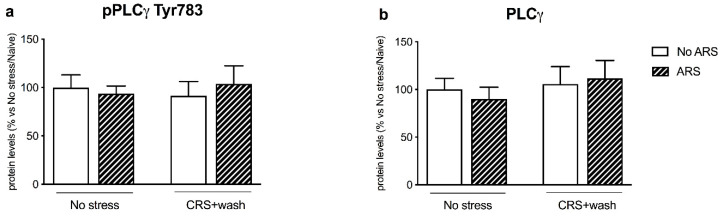
Analysis of PLCγ (phosphorylated and total) (panels **a**,**b**) protein levels in the crude synaptosomal fraction of the prefrontal cortex of chronically stressed rats (CRS) exposed to an acute challenge (ARS) after 3 weeks of washout (wash). The data, expressed as a percent change of No stress/No ARS animals, are the mean ± SEM.

Finally, we did not find any modulation induced by both chronic and acute stressors to the PLCγ pathway (Figure 6a,b) in the crude synaptosomal fraction.

## 3. Discussion

In this work, we demonstrated that chronic restraint stress produces protracted molecular changes that impair the activation of BDNF signaling following a subsequent acute stressor and inhibit the ability of PFC to cope with an acute challenge.

Stress is widely recognized as the major environmental factor causing psychopathologies and, in particular, depression [26]. Despite the majority of depressed patients potentially achieving remission following successful pharmacological treatment, there is a high percentage of them that experience relapse, usually as a consequence of environmental adversities, underlying the need to identify novel pharmacological targets to prevent the relapse.

In this field, the investigation of the role of plasticity in the adult rodent brain in the aftermath of chronic stress is growing.

On these bases, we investigated how chronic stress may affect the ability of the prefrontal cortex to react to a subsequent hit in the rest period, in order to identify long-lasting stress-induced changes.

In line with our previous data [23,24], exposure to chronic stress induced cognitive deficits in adult male rats. This negative effect of stress may be responsible for the alteration in the functionality of the HPA axis; indeed, we observed that ARS increased the levels of circulating CORT in control animals, a modulation that was inhibited in animals exposed to the challenge following the rest period.

Moreover, after three weeks of washout from stress, the activity-dependent genes *Arc* and *cFos* were significantly decreased in the PFC of stressed rats, an effect that was previously observed after 11 days of the chronic restraint protocol [27]. In addition, we found that stress exposure persistently reduced *Gadd45β* expression with respect to control animals, in line with the downregulation of *Gadd45β* in the PFC that has been formerly detected following exposure to the chronic mild unpredictable stress protocol [28]. Together, these data suggested that the effect of stress exposure on the gene expression of the IEGs was long-lasting.

However, when we measured their expression following the subsequent exposure to the acute challenge, we observed a rapid and strong increase in the IEGs, independently from CRS, indicating that the PFC preserves a functional plasticity after the post-stress period, as it adequately reacted to a subsequent acute stress exposure in terms of *Arc*, *cFos*, and *Gadd45β* gene expression. In line, the PFC is a brain region with high levels of structural and functional plasticity that permits itself to modify brain functioning by internalizing behavioral experiences [20]. Accordingly, Moench and colleagues demonstrated that elevated platform stress increased *cFos* expression compared to control animals, although this increase was attenuated when the animals were previously exposed to CRS [29].

With regard to neuroplasticity, it has been effectively demonstrated the dichotomic influence of stress on BDNF with the detrimental effect due to chronic exposure [19,30,31], with a positive modulation exerted by an acute challenge [22,32,33,34,35].

Here, we demonstrated that ARS induced an upregulation of mBDNF and of its receptor TRKB protein levels, whereas these modulations were completely blunted in rats previously exposed to CRS.

Accordingly, the inability to activate the BDNF-TRKB pathway in stressed animals was reflected by the blunted induction of their intracellular-related pathways. Indeed, one hour of acute restraint stress upregulated pAKT Ser473 and the downstream effector pS6 Ser240/244 in non-stressed animals, as we previously found in rats exposed to 5 min of forced swim stress (FSS) [34]. Moreover, acute stress enhanced the protein expression of pERK1/ERK1 and pERK2/ERK2 specifically in control animals, as reported by Shen and colleagues following FSS [36], results that were, again, missed in stressed rats. By contrast, the PLCγ pathway was not activated by the acute challenge both in non-stressed and stressed rats, suggesting specificity in the signaling activated by acute restraint stress.

The observation that the levels of mBDNF and its downstream effectors were similar to non-stressed animals after 21 days of stress washout was already published by others groups [37,38]. As several studies showed that BDNF was reduced immediately after the stress exposure [19,30,31], our findings suggested that the modulation of BDNF signaling was returned to baseline after the rest period. Accordingly, many studies provide evidence that there is a reversibility in dendritic architecture following a period of rest from chronic stress in the PFC and in the Hip [39].

However, the failure to activate BDNF cascades in response to acute stress in CRS animals indicated that the system is not completely recovered from the effects induced by chronic stress, but that some molecular scars, that inhibit the recruitment of the adaptive mechanisms activated in healthy subjects, are still present. By note, as we recently demonstrated that ARS induced an improvement in the cognitive performance by positively modulating BDNF expression in the PFC [22], we could speculate that the inhibition of BDNF signaling in animals previously stressed may suggest the persistence of the cognitive deficits in the CRS group.

Finally, we are aware that this study presents some limitations. First, the molecular analyses were conducted at only one time point (one hour) after the ARS, timing that has been chosen on the bases of our previous results. Indeed, we recently demonstrated that *Bdnf* gene expression was specifically modulated in the PFC of control animals in the time frame of one hour following ARS [22]. Similarly, we decided to deeper investigate the activation of BDNF signaling specifically in the PFC, and not in the dorsal and ventral Hip, based on our previous results obtained in this brain region after the exposure to the ARS [22], even if it is known that the whole Hip is involved in the modulation of stress response. Lastly, the cognitive task is evaluated by employing only a single behavioral test, the novel object recognition test that is, however, widely used to measure working memory, a process strictly connected with the PFC [40].

## 4. Materials and Methods

### 4.1. Animals

Adult male Sprague–Dawley rats (Charles River, Calco, Italy) (*n* = 40) were grouped-housed with food and water freely available and were maintained under standard animal facility conditions (constant temperature of 22 ± 2 °C, 12 h light/dark cycle, humidity of 50 ± 5%). Animals were acclimated for two weeks prior to the starting of the experiment. The experimental protocol has conformed to the rules and principles of the 2010/63/UE Directive, and was approved by the Italian Health Ministry (authorization *n* 151/2017-PR). This study research complies with the commonly accepted ‘3Rs.’

### 4.2. Stress Procedure and Behavioral Test

After two weeks of adaptation to the housing conditions, rats were divided into two groups (5–10 animals for groups): Non-stressed and stressed animals. During the CRS paradigm, stressed rats were exposed two times/day for four weeks to one hour of restraint stress at random hours, to avoid habituation. After each session of restraint stress, animals were grouped-housed in their home cages. The dimensions of the restrainer were similar to the size of the animal, which made the animal almost immobile in it. In addition, 24 h after the last stressor, animals were tested in the NOR task as previously described [22,23,24]. Briefly, the test consists of three phases: The encoding phase of 5 min with two identical objects in an arena, the consolidation phase of 1 h in the home cage, and the retrieval phase of 5 min, in which one of the objects presented previously was replaced by a new one. Discrimination index was evaluated according to the formula: Time of novel object minus time of familiar object exploration divided by time of novel plus familiar object exploration, multiplied by 100. After 4 weeks of CRS, animals underwent a period of three weeks of washout from the stress procedure and, following this period, rats were exposed to one hour of ARS and sacrificed one hour later. The prefrontal cortex, including Cg1, IL, and PrL subregions, (Figure S2) corresponding to plates 6–10 of the atlas of Paxinos and Watson [41], was dissected from 2 mm thick slices.

### 4.3. Measurement of Plasma Corticosterone Levels

Trunk blood samples for each rat were collected in tubes with ethylenediaminetetraacetic acid and were centrifuged for 20 min at 3000 rcf and 4 °C for the separation of the plasma. An enzyme-linked immunosorbent assay was employed to determine the corticosterone plasma levels, according to the manufacturers’ instructions (IBL international).

### 4.4. RNA Preparation and Gene Expression Analysis by Quantitative Real-Time PCR

Total RNA was isolated using PureZol RNA isolation reagent (Bio-Rad Laboratories, Italy) as previously reported [42]. The RNA concentrations were measured by spectrophotometry (OD260/280 1.8 < ratio < 2). The samples (10 ng/µL) were processed for real-time polymerase chain reaction (RT-PCR) to assess *Arc*, *Gadd45*β, and *cFos* using the iScriptTM one-step RT-PCR kit for probes (Bio-Rad Laboratories, Segrate, Italy) and analyzed with the TaqMan qRT PCR CFX384 instrument of Bio-Rad Laboratories. Samples were run in 384-well formats in triplicate as multiplexed reactions with the *36b4* gene as internal control. Primer sequences (Table 2A/B) used were purchased from Eurofins MWG-Operon and Life Technologies.

Thermal cycling was initiated with an incubation at 50 °C for 10 min (RNA retrotranscription) and then at 95 °C for 5 min (TaqMan polymerase activation). After this initial step, 39 cycles of PCR were performed. Each PCR cycle consisted of heating the samples at 95 °C for 10 s to enable the melting process, and then for 30 s at 60 °C for the annealing and extension reactions. A comparative cycle threshold method was used to calculate the relative target gene expression by applying the 2^−∆(∆CT)^ method [43].

### 4.5. Protein Extraction and Western Blot Analysis

mBDNF, TRKB, phospho AKT Ser473, AKT, phospho S6Ser240/244, S6, phospho ERK1 Thr202, ERK1, phospho ERK2 Tyr204, ERK2, phospho PLCγ Tyr783, and PLCγ protein levels were assessed by Western blot analysis. Tissues were manually homogenized and centrifuged to obtain the subcellular fractions as previously described [44] (see [32] for the purity of the fraction obtained). The Bradford Protein Assay procedure (Bio-Rad Laboratories, Segrate, Italy) was employed to measure the total protein content with bovine serum albumin as a calibration standard. In addition, 10 µg of protein was run under reducing conditions using Tris-Glycine eXtended precast gels (Bio-Rad Laboratories) and then electrophoretically transferred onto nitrocellulose membranes (Bio-Rad Laboratories). The membranes were blocked with 10% milk powder and the incubation with the primary antibodies (see Table 2) was carried out overnight. Blots were then incubated for 1 h at room temperature with the opportune secondary antibody, as summarized in Table 3.

Immunocomplexes were visualized by chemiluminescence using the Western Lightning Plus ECL (PerkinElmer, Milano, Italy) and the Chemidoc MP imaging system (Bio-Rad Laboratories, Segrate, Italy). Results were standardized using β-ACTIN as the control protein, which was detected by evaluating the band density at 43 kDa. The Western blot bands are reported in Figure S3.

### 4.6. Statistical Analyses

The results of the behavioral test were analyzed with the Unpaired-T test. The effects of the independent factors CRS and ARS were analyzed with the two-way analysis of variance (ANOVA: ANALYSIS OF VARIANCE), and further differences, when appropriate, were analyzed by Fisher’s protected least significance difference (PLSD: protected least significance difference). Significance for both the behavioral and molecular results was assumed for *p* < 0.05.

## 5. Conclusions

In conclusion, the lack of modulation of the BDNF-related cascades induced by ARS in CRS animals could indicate a long-lasting effect of chronic stress exposure on mechanisms of synaptic plasticity within the PFC, thus underlying how the detrimental consequences of stress may be unmasked by the exposure to a second hit.

These results reveal the complexity of the neuroplastic machinery set in motion to cope with challenges, mainly when the system was previously impaired, adding critical new information useful for promoting adaptive plasticity and resilience.

## Figures and Tables

**Table 1:** Analysis of TRKB receptor (full-length and truncated form) protein levels in the whole homogenate of the prefrontal cortex of chronically stressed rats (CRS) exposed to an acute challenge (ARS) after 3 weeks of washout (wash). The data, expressed as a percent change of No stress/No ARS animals, are the mean ± SEM.

	No Stress/No ARS	No Stress/ARS	CRS/No ARS	CRS/ARS
TRKB fl	100 ± 9	78 ± 17	117 ± 10	91 ± 18
TRKB tr	100 ± 14	79 ± 19	103 ± 7	77 ± 9

**Table 2:** A–B: Primers and probes purchased from Eurofins MWG-Operon (**A**) and probes purchased from Life Technologies (**B**).

**(A)**	**Forward Primer**	**Reverse Primer**	**Probe**
*Arc*	GGTGGGTGGCTCTGAAGAAT	ACTCCACCCAGTTCTTCACC	GATCCAGAACCACATGAATGGG
*cFos*	TCCTTACGGACTCCCCAC	CTCCGTTTCTCTTCCTCTTCAG	TGCTCTACTTTGCCCCTTCTGCC
*36b4*	TCAGTGCCTCACTCCATCAT	AGGAAGGCCTTGACCTTTTC	TGGATACAAAAGGGTCCTGG
**(B)**	**Accession Number**	**Assay ID**	
*Gadd45*β	BC085337.1	Rn01452530_gI	

**Table 3:** Antibodies condition used in the Western blot. Milk: M, overnight: O/N, room temperature: RT, bovine serum albumin: BSA.

	Primary Antibody	Secondary Antibody
mBDNF (14kDa)	1:1000 M 3% (Icosagen), 4° O/N	Anti-mouse1:3000 M3%, 1h RT
TRKB (145–95 kDa)	1:750 M5% (Cell signaling), 4° O/N	Anti-rabbit 1:2000 M5%, 1h RT
TRKB Tyr 706 (145 kDa)	1:500 M5% (Cell signaling), 4° O/N	Anti-rabbit 1:1000 M5%, 1h RT
AKT Ser473 (60 kDa)	1:1000 M5% (Cell signaling), 2h RT	Anti-rabbit 1:1000 M5%, 1h RT
AKT (60 kDa)	1:1000 M10% (Cell signaling), 4° O/N	Anti-rabbit 1:1000 M5%, 1h RT
pS6 Ser240/244 (32 kDa)	1:1000 M5% (Cell signaling), 4° O/N	Anti-rabbit 1:2000 M5%, 1h RT
S6 (32 kDa)	1:1000 M5% (Cell signaling), 4° O/N	Anti-rabbit 1:2000 M5%, 1h RT
pERK1/2 Thr202/ Tyr204 (44 kDa)	1:1000 M3% (Cell signaling), 4° O/N	Anti-rabbit 1:2000 M3%, 1h RT
ERK1/2 (44 kDa)	1:1000 M3% (Santa Cruz Biotechnology), 4° O/N	Anti-rabbit 1:5000 M3%, 1h RT
pPLCγ Tyr783 (155 kDa)	1:1000 BSA5% (Cell signaling), 4° O/N	Anti-rabbit 1:2000 M5%, 1h RT
PLCγ (155 kDa)	1:1000 M5% (Cell signaling), 4° O/N	Anti-rabbit 1:1000 M5%, 1h RT
β-ACTIN (43 kDa)	1:10,000 M3% (Sigma-aldrich), 4° O/N	Anti-mouse 1:10,000 M3%, 1h RT

## Supplementary Materials

Supplementary materials can be found at https://www.mdpi.com/1422-0067/21/17/6261/s1. Figure S1: Analysis of the cognitive performance in chronically stressed (CRS) rats. The data are the mean ± SEM. * *p* < 0.05 vs No stress (Unpaired T-test). Figure S2: Schematic representation of the subregions (Cg1, PrL and IL) of the PFC considered for the molecular analysis (plate 6–10 of Paxinos [42]). Figure S3: Western blots bands and related experimental groups. 1–9: No stress/No ARS; 12–17: No stress/ARS; 61-70: CRS+wash/No ARS; 71–80: CRS+ wash/ARS.
