# Supplementary material for: Chronic Restraint Stress Inhibits the Response to a Second Hit in Adult Male Rats: A Role for BDNF Signaling
Source: Int J Mol Sci. 2020 Aug 29;21(17):6261. doi: 10.3390/ijms21176261 (PMC7503736; doi:10.3390/ijms21176261)

International Journal of
Molecular Sciences

MDPI

Article

Chronic Restraint Stress Inhibits the Response to a Second Hit in Adult Male Rats: A Role for BDNF Signaling

Paola Brivio [1], Giulia Sbrini [1], Giulia Corsini [1], Maria Serena Paladini [2], Giorgio Racagni [1], Raffaella Molteni [2] and Francesca Calabrese [1,*].

[1] Department of Pharmacological and Biomolecular Sciences, Università deglI Studi di Milano, 20133 Milan, Italy; paola.brivio@unimi.it (P.B.); giulia.sbrini@unimi.it (G.S.); giuliacorsini892@gmail.com (G.C.); giorgio.racagni@unimi.it (G.R.)

[2] Department of Medical Biotechnology and Translational Medicine, Università degli Studi di Milano, 20133 Milan, Italy; maria.paladini@unimi.it (M.S.P.); raffaella.molteni@unimi.it (R.M.)

* Correspondence: francesca.calabrese@unimi.it

Received: 3 August 2020; Accepted: 26 August 2020; Published: 29 August 2020

Supplementary figure

Figure S1. *Analysis of the cognitive performance in chronically stressed (CRS) rats.* The data are the mean ± SEM. *p<0.05 vs No stress (Unpaired T-test).

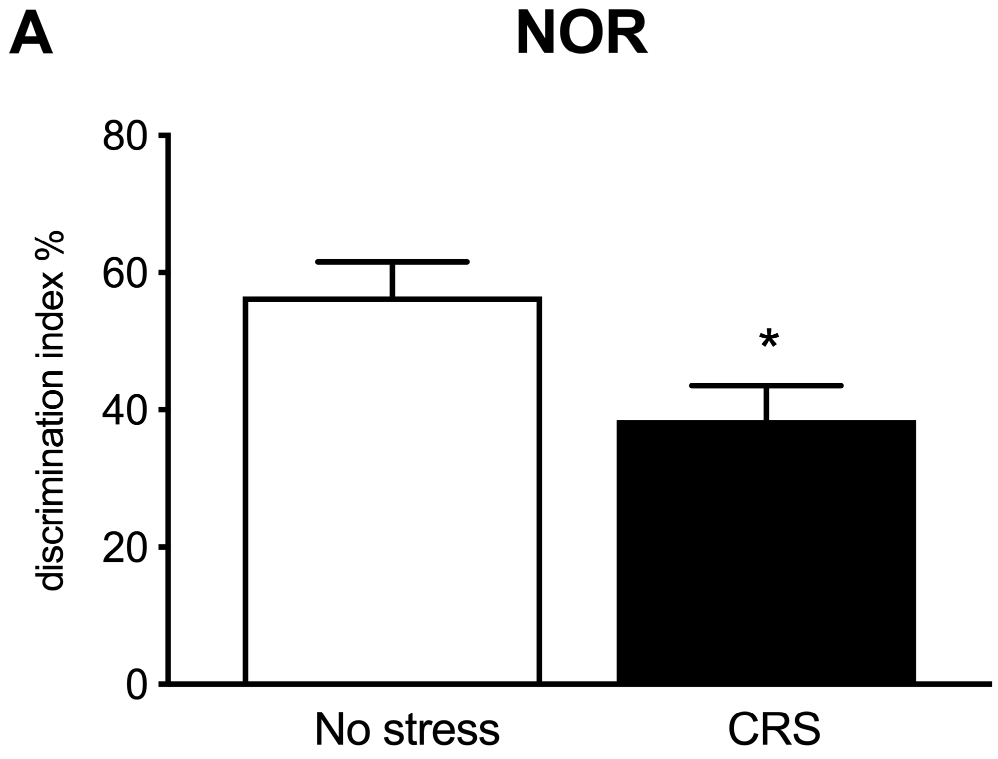

Figure S2. *Schematic representation of the subregions (Cg1, PrL and IL) of the PFC considered for the molecular analysis (plate 6-10 of Paxinos [42]).*

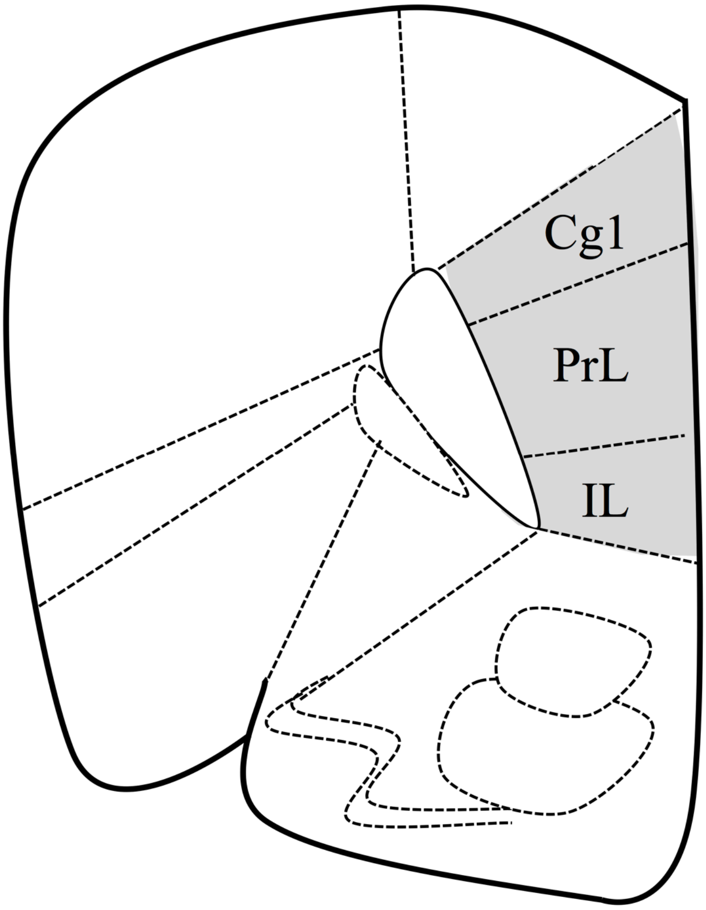

Figure S3. *Western blots bands and related experimental groups.* 1-9: No stress/No ARS; 12-17: No stress/ARS; 61-70: CRS+wash/No ARS; 71-80: CRS+wash/ARS.

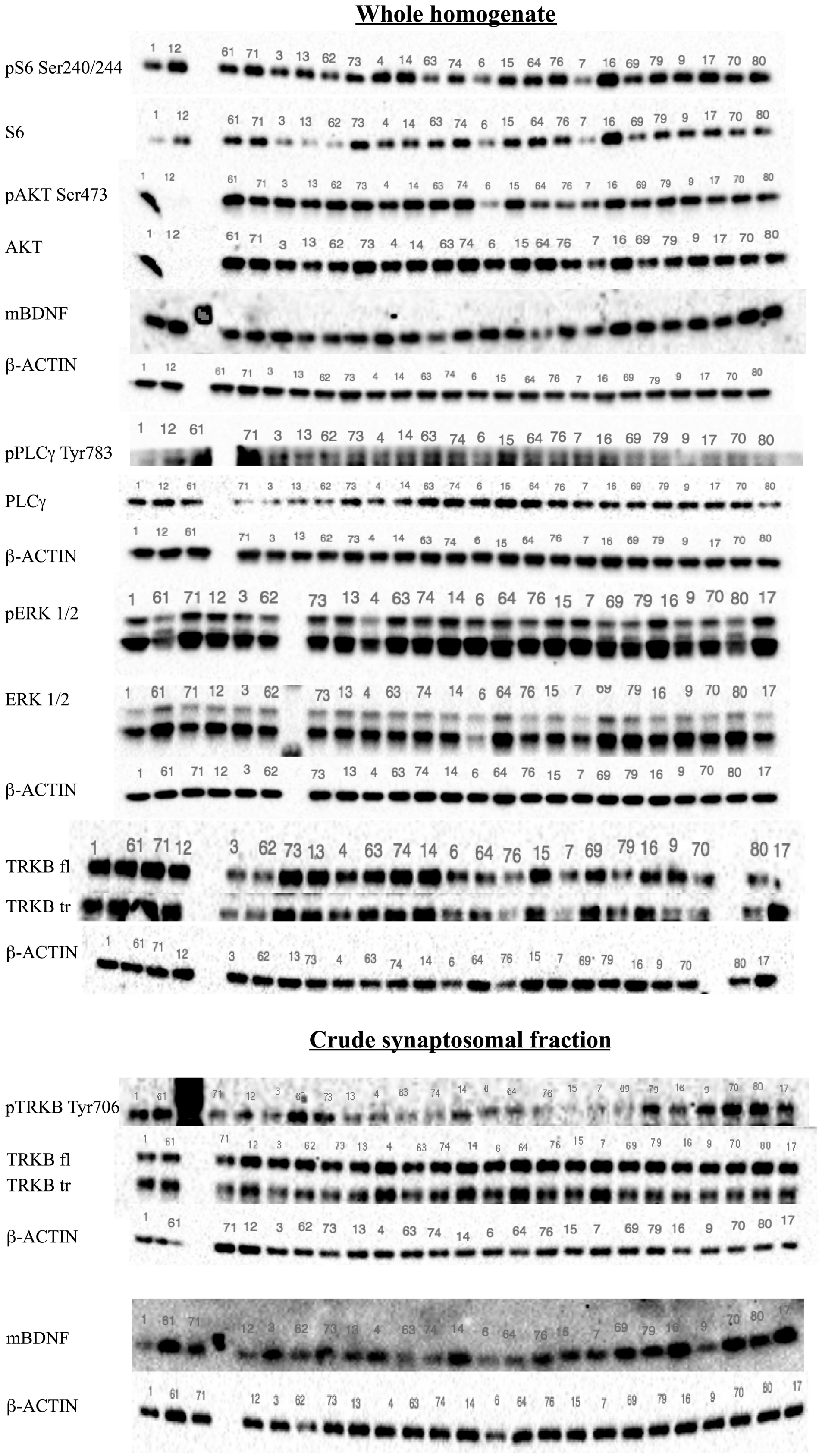

Supplement: Supplementary file 1 [file ijms-21-06261-s001.pdf]
